# Morphological knowledge and spelling in French-speaking adults with developmental dyslexia

**DOI:** 10.1007/s11881-026-00373-2

**Published:** 2026-05-19

**Authors:** Estelle Ardanouy, Pauline Quémart

**Affiliations:** 1 School of psychology, University of Dundee, Dundee, DD14HN Scotland, UK Scrymgeour building,; 2 Laboratoire de psychologie des Pays de la Loire (LPP), Nantes Université, Univ Angers, Nantes, France

**Keywords:** Spelling, Dyslexia, Morphology, Compensation

## Abstract

Spelling errors are among the most persistent symptoms of dyslexia in adults. This study investigated the influence of morphological information on spelling accuracy in French-speaking adults with developmental dyslexia. Specifically, we aimed to (1) compare university-level adults with dyslexia and age-matched controls in spelling morphologically complex words, and (2) examine the role of morphological awareness in spelling among individuals with dyslexia. In total, 53 adults with dyslexia and 55 typical adult readers completed a spelling task with five types of words: words with silent final letters that were either morphologically predictable or not, suffixed words, prefixed words, and inconsistent monomorphemic words. Additional measures included reading fluency, phonological awareness, morphological awareness, vocabulary, and general spelling. Results showed that adults with dyslexia benefited more than controls from morphologically predictable cues in spelling, particularly for silent final letters. Moreover, morphological awareness predicted spelling in adults with dyslexia, even after controlling for phonological awareness and vocabulary. These findings allow us to better understand the spelling profile of adults with dyslexia at university level.

**Supplementary Information:**

The online version contains supplementary material available at https://doi.org/10.1007/s11881-026-00373-2.

## Introduction

Spelling is an essential predictor of academic success at all school levels and higher education (Daffern et al., 2017; Graham & Harris, 2005; Preiss et al., 2013) - as well as of professional achievement, social integration (Martin-Lacroux & Lacroux, 2017) and self-esteem (Riddick et al., 1999). However, it is one of the most challenging abilities for children and adults to develop, even more so than reading, because it requires a number of additional skills over and above those required by reading. Spelling places greater demands on working memory, requires mastery of handwriting movements and is more complex in language such as French and English due to its orthographic inconsistency. Because of this complexity, spelling difficulties are common in adults with dyslexia (Reis et al., 2020), particularly in inconsistent orthographies (Daigle et al., 2016; Giannouli & Pavlidis, 2014). These difficulties are acknowledged in the latest definitions of dyslexia (Carroll et al., 2025; International Dyslexia Association, 2025). Spelling often remains more severely impaired than reading (Moll & Landerl, 2009) even in high-functioning adults with dyslexia (Kemp et al., 2009; Lefly & Pennington, 1991) who struggle to detect their own errors (Mazur & Chenu, 2023). Improving spelling outcomes for these students is therefore of high importance. In this study, we look at the influence of morphological information on spelling accuracy in adults with dyslexia.

###  Morphological information in the French orthographic system

In alphabetic writing systems, learning to spell starts by mapping phonemes to graphemes. Yet, mastering spelling extends beyond this process, particularly in deep orthographies where learners must cope with numerous inconsistencies. The French orthographic system is consistent in terms of reading but inconsistent in terms of spelling. Ziegler et al. (1996) estimate that a French monosyllabic word has a 79% chance of having multiple possible spellings. In many cases, these inconsistencies can be overcome by relying on morphological information – that is, cues from the internal structure of a word, such as roots, affixes and morphologically predictable silent letters.

Morphological information can support accurate spelling in several ways. First, it can help represent features of written language that have no phonological counterpart. One of the specific features of French orthography concerns final silent letters. According to the *Silex* database, 28% of French words end with a silent final letter (Gingras & Sénéchal, 2017). These final silent letters can have a morphological justification, which aids in memorizing them (Pacton & Deacon, 2008). For example, in the word “renard”/ʁənaʁ/[fox], the “d” is silent but can be predicted from related words where the “d” is pronounced: “renarde”/ʁənaʁd/[vixen], “renardeau”/ʁənaʁdo/[fox cub]. Second, inconsistencies in phoneme-grapheme correspondence in French language can also be overcome by applying the principle of morphological consistency. Many inconsistent bigrams or trigrams can be more easily written if identified as part of morphological units. For example, the suffix *-tion* in French marks an action, as in “creation” [creation], and helps eliminate the use of other spellings such as *-sion*,* -ssion*,* -cion*,* -xion*. Overall, these features of the French system explain why identifying oral and/or written morphological information should facilitate spelling.

### Morphological knowledge and spelling theories

The ability to identify and use morphological information when processing oral and written language falls under the concept of morphological knowledge, an umbrella term that encompasses the ability to process morphemes, whether explicitly—often referred to as morphological awareness—or implicitly (Bowers et al., 2010; Goodwin et al., 2021). This concept encompasses both the manipulation of morphological units in spoken language and the processing of morphemes in reading and spelling (Goodwin et al., 2021).

From a developmental perspective, the ability to identify and use morphemes when spelling – a particular domain of morphological knowledge – emerges early (Ardanouy et al., 2024; Treiman & Kessler, 2014). While early models of spelling acquisition viewed reliance on morphological information as a marker of expertise, more recent theoretical frameworks suggest that morphological knowledge can develop even in individuals with poor phonological skills.

Several accounts predict that phonological, orthographic, and morphological knowledge are integrated from the earliest stages of spelling development (Berninger et al., 2010; Levesque et al., 2021; Treiman & Kessler, 2014). The Triple Word Form theory emphasizes the interdependent development of orthographic, phonological, and morphological knowledge, each contributing complementarily to spelling acquisition (Berninger et al., 2010; Richards et al., 2006). Similarly, the Integration of Multiple Patterns (IMP) framework proposes that learning to spell involves the integration of multiple sources of information, including phonological, morphological and graphotactic patterns^1^, which children gradually acquire to produce accurate spellings (Treiman, 2017; Treiman & Kessler, 2014). This perspective aligns with connectionist models, which further suggest that spelling emerges from the gradual learning of statistical regularities in the mappings between orthography, phonology, and semantics, rather than from a strictly sequential reliance on distinct representational units (e.g., Harm & Seidenberg, 2004; Plaut et al., 1996). Together, these frameworks support the view that morphological knowledge can develop in parallel with other forms of linguistic knowledge and may provide an additional pathway to support spelling, even in individuals with weak phonological skills. This theoretical point is key to understanding spelling skills in individuals with phonological deficits, in particular in dyslexia (Cavalli et al. 2017; Georgiou et al. 2023; Law et al. 2015).

### Morphological knowledge and its predictive role in spelling in individuals with dyslexia

Longitudinal studies have highlighted the predictive role of morphological awareness, a particular domain of morphological knowledge on spelling acquisition across several languages, including English (Deacon et al., 2009), French (Desrochers et al., 2018) and Greek (Manolitsis et al., 2019). Morphological awareness is a multidimensional construct typically assessed through a range of metalinguistic tasks requiring individuals to consciously reflect on and manipulate morphemic units within words, such as producing derived forms, or deciding whether or not a word is affixed (Goodwin et al., 2017; Tighe & Schatschneider, 2015). Research in typically developing readers suggests that the predictive role of morphological awareness on spelling is partly independent of phonological skills (Deacon et al., 2009; Desrochers et al., 2018). This raises the question of whether individuals with dyslexia can similarly draw on morphological awareness to support their spelling, despite their persistent phonological deficits.

Several studies have looked at how morphological awareness develops in dyslexic children and adolescents. In a meta-analysis conducted across several languages, children with dyslexia showed a level of morphological awareness comparable to that of children matched for reading age (Georgiou et al., 2023). Nevertheless, performance varies across assessment tasks, as suggested by Casalis et al. (2004). Other studies have looked at whether morphological awareness is related to spelling in adolescents with dyslexia. In English, Carlisle (1987) found that ninth-grade students with dyslexia exhibited morphological awareness comparable to that of sixth graders, whereas their spelling performance match that of fourth graders. Thus, their morphological awareness is not sufficient to support spelling performance at a level commensurate with their grade. Similarly, Schiff and Levie (2017) showed that among Hebrew-speaking children, adolescents, and adults with dyslexia, morphological awareness was not a predictor of spelling morphologically derived words, whereas it was for the control group. In summary, children and adolescents with dyslexia appear to show lower morphological awareness than their age-matched peers, although their performance is relatively preserved compared with reading and spelling skills.

Studies investigating adults with dyslexia have examined whether they can catch up with the morphological delay observed in childhood, but findings remain inconclusive. Overall, preserved versus impaired morphological awareness in adults with dyslexia depends on multiple factors. First, task type appears critical: studies using suffixation decision or suffixed word detection tasks often find performance comparable to age-matched controls (Cavalli et al. 2017; Martin et al. 2014), whereas more complex tasks, such as sentence completion or prefixed word detection, tend to reveal persistent deficits (Law et al., 2015, 2018; Martin et al., 2014). Even when accuracy is preserved, adults with dyslexia frequently show slower response times, suggesting increased processing demands or reliance on more effortful strategies (Cavalli et al. 2017; Martin et al. 2014). Language differences may also play a role, as the transparency of the orthographic system and morphological complexity influence task difficulty. For example, in French, adults with dyslexia often perform close to age-matched controls (Cavalli et al. 2017; Martin et al. 2014), while in Dutch and English, morphological awareness deficits are more frequently observed (Law et al., 2015, 2018). Sample characteristics, particularly literacy level and compensation status, further modulate outcomes: adults with dyslexia who have achieved higher literacy performance (‘compensated’) generally perform at the level of age-matched controls, while those with ongoing reading difficulties (‘non-compensated’) show lower morphological awareness (Law et al., 2015). Finally, individual cognitive profiles, including the severity of phonological impairment and vocabulary knowledge, are likely contributors to variability across studies (Carioti et al., 2021; Inoue et al., 2023; Leinonen et al., 2001; McBride-Chang et al., 2005, 2008).

Finally, little is known about the contribution of morphological awareness to spelling in adults with dyslexia. While morphological awareness significantly predicts or is related to spelling in English-speaking poor readers (Cheema et al., 2023; Fracasso et al., 2016), this relationship has rarely been examined in adults with developmental dyslexia. To date, evidence is limited to a small number of studies, most notably Law et al. (2015), who showed that morphological awareness, assessed through a derivational suffix production task and a nonword sentence completion task, significantly predicted word spelling accuracy (standardized test) in English-speaking adults with dyslexia, even after controlling for phonological awareness and vocabulary.

### Influence of morphological information on spelling in typically developing readers

The ability to identify written morphological units in print also facilitates spelling. Children are more accurate in spelling derived words compared to non-derived words (Casalis et al., 2011; Deacon & Bryant, 2006; Kemp, 2006). For example, in a study by Deacon and Bryant (2006), English-speaking children aged 7 to 9 used root words (e.g., *turn*) as clues to spell a morphologically related word (e.g., *turning*) more accurately than in a control condition with monomorphemic words (e.g., *turnip*). Results in the same vein were found in French for words with silent final letters. From grade 2 to 4, words are spelled better when a morphological justification exists for the silent final consonant (e.g., the t in “chat” [ʃa]/cat, is predicted by “cha**t**on”/kitten) compared to words without such support (e.g., “brebis” [bʁəbi], ewe) (Sénéchal, 2000; Sénéchal et al., 2006).

Adults also spell word endings more accurately when they are part of a suffix than when they are not (Heyer, 2021; Treiman et al., 2021). In the English language, the probability of spelling the phoneme/əs/with the letters “ous” is higher when it is used as a suffix in an adjective (e.g., famous) than when it is used in a noun (e.g., bonus). Heyer (2021) showed that adults are sensitive to this principle, using the ending -ous to transcribe the phoneme/əs/more often when it functions as an adjective than as a noun. Treiman et al. (2021) confirmed this result and added that the better the participants’ spelling, the more they rely on morphological information. Altogether, these results indicate that children and adults with typical reading development identify morphological units in written words and use morphological consistency to improve the accuracy of spelling inflected or derived forms.

### Influence of morphological information on spelling in individuals with dyslexia

Other studies have shown that individuals with dyslexia also benefit from the presence of morphological units when spelling. However, most of these studies have been conducted in children and have yielded contradictory results. Children with dyslexia spell words representing morphological information better than those that do not (Angelelli et al., 2017; Bourassa et al., 2006; Bourassa & Treiman, 2008; Quémart & Casalis, 2017; Suárez-Coalla et al., 2017). In a spelling-to-dictation task, Italian-speaking third graders with dyslexia were less accurate than age-matched typically developing children, but they spelled morphologically complex words (e.g. “bestiale”, beastly) better than simple words (e.g. “insulso*”*, vapid) (Angelelli et al., 2017). By contrast, Carlisle (1987) failed to show an advantage for morphologically complex words. When asked to spell pairs of words from the same word family (magic/magician), children with dyslexia were more likely than younger controls to misspell one word in the pair, usually the base word. This result suggests that children do not systematically apply the principle of morphological consistency. Similarly, Bourassa et al. (2019) found that children and adolescents with dyslexia benefit from morphological information when spelling, similar to younger peers matched on spelling level but less than children of the same chronological age.

As noted above, one of the peculiarities of French spelling is the large number of silent letters at the end of words. These silent letters can often be inferred from the activation of words in the same morphological family. A study investigated the spelling of these letters in French-speaking adolescents with dyslexia, matched with a control group based on reading level (Quémart & Casalis, 2017). The participants were asked to spell two types of words: words with a final silent letter predictable by morphology or not predictable by morphology. The dyslexia group was less accurate than the control group in spelling the final letter. Furthermore, the control group performed similarly for both types of words (predictable by morphology or not), while the dyslexia group benefited more from the condition where the final letter was predictable by morphology. This result indicates that adolescents with dyslexia rely more on morphological information than younger children matched by reading level.

When focusing on the spelling skills of adults with dyslexia, the number of studies becomes sparse. Bruck (1993) compared the spelling performance of English-speaking university adults with dyslexia to control groups of the same chronological age or reading level. The dyslexia group performed lower than both control groups on all spelling measures (regular words, irregular words, words whose spelling can be deduced by orthographic rules, words whose spelling can be deduced by morphological rules) except for the spelling of morphologically complex words. In this case, adults with dyslexia achieved similar results to younger reading-level-matched controls. Coleman et al. (2009) also found that English-speaking university-level adults with dyslexia have difficulties in processing the morphological structure of written words compared to same-age adults. They were asked to complete a 30-minute essay and a standardized word spelling task. For the essay, the dyslexia group primarily made phonological and morphological errors at all levels of word complexity, based on word frequency (see also Moats, 1996). The dyslexia group made around 30% morphological errors compared to 15% in the control group (mostly at the derivational level). In the spelling task, the dyslexia group was less effective at using morphological information than the controls. Note, however, that this result should be considered with caution as difficulties on only three words contributed to the significant difference between the dyslexic and control groups in the spelling task. Results from these two studies indicate that adults with dyslexia use morphological information as effectively as younger adolescents matched on reading level, but less effectively than control adults matched on chronological age.

### Current study

Spelling is a major area of difficulty for individuals with dyslexia. However, their morphological knowledge is relatively preserved, as evidence with their ability to perform morphological awareness tasks and to process morphological information when spelling (Angelelli et al., 2017; Bourassa et al., 2006, 2019; Bourassa & Treiman, 2008; Quémart & Casalis, 2017; Suárez-Coalla et al., 2017). Surprisingly, most studies on the role of morphology in spelling in relation to dyslexia have focused on children or adolescents with dyslexia. Only a handful of studies have been conducted in adults (Bruck, 1993; Coleman et al., 2009) and they did not specifically target the spelling of morphologically derived words but rather general spelling skills. Furthermore, to our knowledge, only one study has explored the contribution of morphological awareness to the spelling of morphologically complex words in adults with dyslexia (Schiff & Levie, 2017). This study did not find an effect of morphological awareness on spelling in the dyslexia group, but that group included children, adolescents, and adults. For this reason, we focus here specifically on a single age group: adults with dyslexia.

The present study aims at better understanding whether and how morphological information influences word spelling in adults with dyslexia. More precisely, the two objectives of the current study are: (1) to compare the spelling of morphological information in university-level adults with dyslexia and chronological age-matched controls and (2) to investigate the contribution of morphological awareness to word spelling in adults with dyslexia.

For the first objective, we explored several categories of words in which morphological information is important for spelling. First, participants had to spell words with predictable silent final letters based on morphology, which were then compared to words with non-predictable silent final letters (e.g., “confus” (embarrassed) [kɔ̃fy] predicted by “confuse” [kɔ̃fyz] vs. “velours” (velvet) [vəluʁ]). Next, participants were required to spell inconsistent prefixed, suffixed, and monomorphemic words. Theoretical models of spelling acquisition posit that orthographic, phonological, and morphological information are gradually integrated and jointly support spelling performance (Berninger et al., 2010; Plaut et al., 1996; Treiman & Kessler, 2014). Within these frameworks, morphological information can strengthen lexical representations by providing converging cues when phonological mappings are weak. Based on this theoretical background, we predicted that adults with dyslexia would have lower spelling scores than controls of the same age, but that they would benefit at least as much from morphological information to improve their spelling accuracy (Bruck, 1993; Coleman et al., 2009). More specifically, we predicted better performance for words with predictable silent letters than for those with non-predictable silent final letters in both groups (Quémart & Casalis, 2017). We also expected better performance for affixed words compared to monomorphemic words in both groups (Deacon et al., 2008). Finally, we explored whether prefixed words were better spelled than suffixed words, or vice versa.

For our second objective, we examined whether morphological awareness contributes to the spelling performance of adults with dyslexia. Previous studies in different languages have shown divergent results regarding the morphological awareness skills of adults with dyslexia, but these skills appear to be better preserved than other areas, such as phonological skills (Cavalli et al. 2017; Law et al. 2015, 2018; Martin et al. 2014). However, much less is known about the extent to which morphological awareness predicts spelling performance in this population. While a few studies have examined the influence of morphological knowledge on spelling in poor readers more generally (Cheema et al., 2023; Fracasso et al., 2016), evidence in adults with developmental dyslexia remains extremely limited. Notably, although Law et al. (2015) showed that morphological awareness predicted spelling performance in English-speaking adults with dyslexia, spelling was assessed only at the word level using standardized spelling measures, without examining spelling in sentences or targeting morphologically derived words specifically. Therefore, we tested the predictive value of morphological awareness, assessed through an oral suffixation decision task, on spelling accuracy specifically within the dyslexia group. This task targets awareness of derivational morphemes independently of orthographic processing. We assume that such task reflects the ability to identify and manipulate morphemic structure, which may support spelling by facilitating the segmentation of words into meaningful units and the application of morphologically driven spelling regularities. Taken together, these considerations support our choice of a suffixation decision task, a relatively low-demand oral task that allows us to focus on morphological knowledge while minimizing potential confounding effects related to task complexity. In addition, we controlled for phonological awareness, given its role in spelling acquisition, and vocabulary, which is known to be closely related to both morphological knowledge and spelling skills (Kirby & Bowers, 2017; Kuo & Anderson, 2006; Ocal & Ehri, 2017). We also assumed an effect of morphological awareness on their overall spelling level. Indeed, the link between morphological awareness and spelling doesn’t seem to be limited to morphologically derived words but extends to general spelling skills. For example, Casalis et al. (2011) showed that morphological awareness in French-speaking children in 3rd and 4th grade correlated with the spelling of both derived and non-derived words. However, we predicted a greater contribution of morphological awareness for words containing morphological information (prefixed words, suffixed words, and final silent letters predictable by morphology), after controlling for phonological awareness, a related skill (Deacon & Kirby, 2004; Singson et al., 2000).

Finally, for exploratory purposes, the impact of morphological awareness on spelling skills was compared in two subgroups of dyslexics: compensated versus non-compensated. The subgroups were created according to the definition given by Cavalli et al., 2017. Compensated profiles were examined in terms of a dissociation between morphological and phonological skills (impaired phonological awareness but preserved morphological awareness). We expected that compensated profiles might show relatively better spelling performance and greater benefit from morphological cues than non-compensated adults, in light with findings from Cavalli et al. (2017) for reading.

## Method

### Participants

A total of 108 French-speaking university-level adults took part in this study. Participants were recruited in France and in Switzerland, through social media, speech and language therapists (via parents of their patients), patient associations, and the personal networks of the students and the first author. Two groups were created: a dyslexia group (*N* = 53; from 19 to 40 years old) and a control group of typical readers (*N* = 55; from 20 to 36 years old), matched for chronological age, vocabulary level, and gender (see Table 1 for the details). To be included in the study, participants were required to have validated at least one year of university education (regardless of current student status) and to be native French speakers. However, 18 participants were multilingual: 11 in the dyslexia group (24% Spanish, 18% English, 12% Italian, 12% Persian, 12% German, 12% Arabic, 5% Luxembourgish, and 5% Portuguese) and eight in the control group (40% English, 20% Dutch, 20% Portuguese, 10% Italian, and 10% German). Participants with uncorrected vision or hearing impairments or neurological disorders were excluded from both groups. All the dyslexics had received a diagnosis of developmental dyslexia in their childhood and had undergone speech therapy during childhood or adolescence. Among adults with dyslexia, 13 participants reported comorbid disorders: 56.25% with attention deficit disorder with or without hyperactivity, 12.5% with high intellectual potential, 12.5% with dyscalculia, and 6.25% with developmental language disorder. No participants in the control group reported any neurological, psychiatric, cognitive, or learning-related disorders. The two groups differed statistically on the reading (accuracy and efficiency), spelling, phonological awareness (accuracy and response time) and morphological awareness (accuracy) tests (see Table 1).

**Table 1:** Characteristics of the participant groups

	Dyslexia Group *N* = 53	Control Group *N* = 55	T-test or χ² value	*p* value
	M (SD)	M (SD)		
Age (years; months)	25;10 (5;1)	24;7 (3;11)	1.43	0.16
Gender (male)	19	21	0.06	0.80
Vocabulary (/60)	35.94 (6.34)	37.75 (5.51)	1.58	0.12
Reading fluency – no. words correctly read (Alouette test)	247.75 (22.29)	260.85 (3.97)	4.21	< 0.001
Reading efficiency (Alouette test)	342.88 (82.27)	492.21 (70.37)	10.15	< 0.001
Morphological awareness				
Accuracy (/48)	34.21 (4.12)	38.38 (2.78)	5.86	< 0.001
Response time (s)	95.65 (44.45)	85.22 (48.95)	1.12	0.25
Phonemic awareness				
Accuracy (/30)	22.49 (6.51)	27.62 (2.53)	5.36	< 0.001
Response time (s)	19.76 (13.97)	12.47 (7.55)	3.36	0.001
General spelling – no. errors (Chronosdictées test)	27.96 (15.50)	7.13 (4.94)	9.34	< 0.001

### Procedure

This study was approved by the University of Geneva research ethics committee. All the participants signed a written consent form. All sessions took place online via the Zoom platform and were recorded for double scoring. Participants wore earphones or headphones. The testing session was conducted individually with an experimenter (a trained speech-language therapy student or the first author) and lasted between 1 h and 1.5 h. The speech-language therapy students were trained to administer the protocol by the first author during a two-hour briefing session. They then had to carry out an initial pilot session, which was reviewed by the first author (thanks to a video recording) to ensure that everything was carried out correctly. Each student received individual feedback to ensure that the testing was conducted in a strictly consistent manner. The tasks were presented in a randomized order, so the sequence varied across participants. However, that spelling tasks were always interspersed with non-writing tasks to prevent prolonged writing, thereby limiting potential fatigue and attentional effects, particularly in the dyslexia group. For the computer-timed tests (phonemic and morphological awareness, see below), we used the PsyToolkit platform (Stoet, 2010, 2017) so participants could complete the task independently on their computer, with screen sharing enabled to allow the experimenter to verify that there were no issues. For the spelling tasks, participants were asked to write by hand on a sheet of paper. Once the testing was completed, they were required to send us a scan or a picture of their work immediately after the session. For the text reading task, the text was presented on a computer screen. Although screen size, resolution, and distance from the screen could vary slightly between participants, testing was always conducted on a computer to ensure adequate visibility of the text.

We performed double scoring on 20% of the reading fluency test but conducted double scoring on all of the spelling and vocabulary tasks, as these can be subject to inter-rater differences. All double scoring resulted in inter-rater agreement above 95%. However, we did not perform double scoring on the morphological or phonological awareness tasks because they were computerized.

### Measures

#### Vocabulary

Vocabulary was assessed using the Vocabulary subtest from the French version of the WAIS-IV (Wechsler, 2008). This subtest involved providing oral definitions for 27 words of increasing difficulty, with one example item being “apple.” An incorrect definition received a score of 0, an incomplete definition received 1 point, and a complete definition received 2 points per well-defined word. The maximum possible score was 60 points (the first 3 items are only administered if there is concern about intellectual disability, which did not apply to the study population). The reliability reported in the manual was α = 0.90. The inter-rater reliability for the double scoring was 96.4%.

#### Reading

The standardized Alouette test (Lefavrais, 2005) involved reading a text of 265 words as quickly and accurately as possible for a maximum of 3 min. Participants could not rely on the context because the text was meaningless and contained rare and tricky words. The test gave two scores: an accuracy score based on the number of words correctly read and a reading efficiency score based on reading time and accuracy, with higher raw efficiency scores indicating better performance. Cavalli et al. (2018) validated the psychometric qualities of this test and provided normative data for French university adults. Standard scores are z-scores computed relative to these normative data, with negative values indicating performance below the normative mean. The dyslexia group obtained an average standard score of −4.09 for accuracy and − 2.58 for efficiency. In contrast, the control group achieved higher standard scores (1.81 and − 0.74, respectively), indicating better reading performance. We performed double scoring for 20% of the participants, achieving an inter-judge agreement of 98.1%.

#### Morphological awareness

We used the computerized suffixation decision task described by Cavalli et al. (2017). To our knowledge, this is the only French morphological awareness task that has been validated and used in adults with dyslexia. The stimuli for this task comprised 24 bisyllabic and 24 trisyllabic words, with half having suffixes (e.g., jeunette [young woman]) and the other half having suffix-like endings (e.g., galipette [somersault]). For each trial, participants had to determine whether the word had a suffix or not. Words were presented orally by the computer. Participants made a rapid decision about each word heard in isolation by pressing a key on their keyboard, following a fixation cross that appeared in the center of the screen. This task yielded two scores: response time and accuracy (maximum of 48 points). The accuracy score is the one that will be used in the analyses. Reliability was α = 0.70.

#### Phonemic awareness

The computerized initial phoneme deletion test was developed by Cavalli et al. (2024). Participants were required to remove the initial phoneme from a pseudoword as quickly and accurately as possible. For example, for the item/tʁa/, they needed to answer/ʁa/. The task consisted of 30 pseudowords with a consonant-consonant-vowel structure. Participants heard the items through their computer. The experimenter pressed the computer keyboard to stop the timing and then used the keyboard to indicate whether the answer said orally was correct or incorrect. This task yielded two scores: response time and accuracy (maximum of 30 points). The accuracy score is the one that will be used in the analyses. Reliability was α = 0.87.

#### Spelling

##### Sentence dictation test

We used a dictation task from the standardized test “Chronosdictées” (Baneath et al., 2015). The participants were required to write eight sentences totaling 124 words. This assessment enabled us to calculate several scores: the number of lexical spelling errors, inflectional errors, phonologically plausible errors, segmentation errors (e.g., incorrect segmentation of the words *jembarquerai/j’embarquerai [I will board]), word omissions, and a total error score. The total error score was particularly useful for differentiating our two groups of participants (see Table 1). The average for the dyslexia group was below the 5th percentile, while that of the control group was around the 50th percentile. We conducted double scoring for all the dictations, achieving an inter-judge agreement of 98.7%.

##### Experimental stimuli

The experimental stimuli from 5-word lists (detailed below) were dictated in a randomized order. Each word was dictated three times: first alone, then within a sentence to provide context, and finally alone again. For example, “Pistolet [pistol]. Il tenait un pistolet à la main [He was holding a pistol in his hand]. Pistolet [pistol]”. An example was given to ensure understanding of the instructions. The inter-rater reliability for the double scoring of all word lists was 97.8%.

##### Words with silent final letter

Two lists of words were built for the experiment, each consisted of 10 words with a silent final consonant. The first list consisted of words with silent final letters that were morphologically predictable (SFLmorpho). For example, the word “rabais”/ʁabɛ/[discount] is predictable from the related word “rabaisser.”/ʁabese/[pull down]. The 10 words in the control condition had silent final letters that were not morphologically predictable (SFLcontrol). The two lists were matched for frequency (*M*SFLmorpho = 7.78; *MSFLcontrol* = 7.32), number of phonemes (*M*SFLmorpho = 4.90; *M*SFLcontrol = 5.40), number of syllables (*MSFLmorpho* = 2.5; *M*SFLcontro*l* = 2.5) and orthographic consistency (*M*SFLmorpho = 0.65; *M*SFLcontrol = 0.61) (New et al., 2004). Two separate scores were calculated: one for the spelling of the whole word, and another for the spelling of the final silent letter. Reliability was the same for both lists, at α = 0.78. The two lists are available in Appendix A.

##### Derived words vs. monomorphemic words

Three other lists of 18 words were created for the experiment. The first list contained complex prefixed words (e.g., **in**nombrable [countless]), the second consisted of complex suffixed words (e.g., exig**ence** [demand]), and the third (control) list included inconsistent monomorphemic words^2^ (e.g. blasphémer [to blaspheme]). The three lists were matched for frequency (*M1* = 10.50, *M2* = 9.00, *M3* = 8.79), number of phonemes (*M*Prefixed = 7.17, *M*Suffixed = 7.39, *M*Monomorphemic = 6.67), number of syllables (*M*Prefixed = 3.33, *M*Suffixed = 3.17, *M*Mononorphemic = 2.78) and orthographic consistency (*M*Prefixed = 0.65, *M*Suffixed = 0.61, *M*Monomorphemi*c* = 0.65) (New et al., 2004). For each condition, three separate scores (each out of 18 points) were computed: one point was awarded for each correctly spelled word, one for each correctly spelled base word, and one for each correctly spelled affix. Reliability was the same for all three lists, at α = 0.75. The three lists are available in Appendix B.

## Results

### Descriptive analyses

Descriptive analyses for all word lists of the experimental stimuli are presented in Table 2. Correlations between the raw score of the different variables are displayed by group (dyslexics vs. controls) in Table 3. In both groups, spelling scores across word types showed moderate to strong correlations (e.g., *r* =.64 between SFLmorpho and prefixed words in the control group). Spelling accuracy for affixed words was significantly correlated with general spelling accuracy, particularly in the dyslexia group (e.g., above *r* = −.49 with prefixed and suffixed words). Morphological awareness showed modest correlations with derived word spelling (*r* =.32 for prefixed words but no significant correlation with suffixed words) in the dyslexia group. Phonemic awareness and vocabulary, by contrast, showed weaker and less consistent associations with spelling performance.

**Table 2:** Descriptive statistics (means, SD) for the spelling word lists of the experimental stimuli

	Dyslexia Group	Control Group
SFLmorpho – score on final letter (/10)	6.52 (2.43)	9.11 (1.05)
SFLmorpho – score on entire word (/10)	5.55 (2.44)	8.51 (1.56)
SFLcontrol – score on final letter (/10)	4.46 (2.11)	7.35 (1.67)
SFLcontrol – score on entire word (/10)	3.08 (2.12)	6.55 (1.81)
Prefixed words (/18)		
Whole word (/18)	9.25 (3.33)	14.56 (2.86)
Prefix (/18)	13.88 (2.47)	16.35 (1.68)
Base (/18)	11.85 (2.69)	15.85 (1.95)
Suffixed words (/18)		
Whole word (/18)	7.81 (2.28)	12.07 (2.52)
Suffix (/18)	15.38 (1.30)	16.82 (0.93)
Base (/18)	8.54 (2.56)	12.71 (2.30)
Inconsistent monomorphemic words (/18)	4.96 (2.81)	10.31 (2.84)

*SFL* Silent Final Letter

**Table 3:** Correlation matrix displaying correlations between all variables, bottom left for dyslexia group and top right for control group

	1	2	3	4	5	6	7	8	9	10
1. SFLmorpho (final letter)		0.53***	0.52***	0.64***	0.34*	− 0.53***	0.09	− 0.02	0.14	0.25†
2. SFLcontrol (final letter)	0.52***		0.56***	0.62***	0.43***	− 0.49***	0.18	0.12	0.18	0.23†
3. Prefixed words (whole word)	0.55***	0.69**		0.71***	0.39**	− 0.43***	− 0.05	− 0.04	0.22	0.17
4. Suffixed words (whole word)	0.54***	0.50***	0.71***		0.31*	− 0.49***	− 0.04	0.09	0.20	0.04
5. Monomorphemic words	0.63***	0.60***	0.63***	0.62***		− 0.44***	− 0.03	0.06	− 0.08	0.34*
6. General Spelling (Chronosdictées)	− 0.61***	− 0.63***	− 0.73***	− 0.49***	− 0.67***		− 0.02	− 0.15	− 0.14	− 0.19
7. Reading efficiency (Alouette)	0.41**	0.32*	0.38**	− 0.04	0.54***	− 0.61***		0.16	0.21	0.19
8. MA (accuracy)	0.24†	0.31*	0.32*	0.09	0.30*	− 0.38**	0.13		0.17	− 0.03
9. PA (accuracy)	0.07	0.16	0.35*	0.20	0.02	− 0.30*	0.01	0.29*		0.27*
10. Vocabulary	0.29*	− 0.07	0.26†	0.28*	0.08	− 0.23†	0.07	0.21	0.11	

*** *p* <.001; ** *p* <.01; * *p* <.05 † *p* <.10; *SFL* Silent Final Letter, *MA* Morphological awareness, *PA* Phonemic Awareness

### Spelling of morphological information in adults with dyslexia

The first aim of the study was to compare the spelling of morphological information in university-level adults with dyslexia and chronological age-matched controls. Spelling accuracy (correct vs. incorrect) was analyzed using generalized linear mixed models with a binomial link function, implemented in the lme4 package (Bates et al., 2015) in R software (version 4.4.1). Two separate analyses were conducted. The first included Group (dyslexic, control) and Type of silent letter (SFLmorpho, SFLcontrol), along with their interaction, as fixed effects. The second included Group (dyslexic, control) and Type of word (prefixed, suffixed, monomorphemic) along with their interaction, as fixed effects. Both models included random intercepts for participants and items to account for variability at both levels. Significance of fixed effects was assessed using Wald z tests. The models did not exhibit multicollinearity among the predictors, as all variance inflation factors (VIFs) were below 2 (James et al., 2017).

#### Words with silent final letters

The first set of analyses was conducted using spelling accuracy for the silent final letter as the dependent variable. Model fit indices are reported for completeness (Log-Likelihood = −994.07; AIC = 2000.14, BIC = 2034.18, Deviance = 1708.23). Table 4 presents the results of the generalized linear mixed model and the corresponding odds ratios derived from the log-odds estimates.

**Table 4:** Generalized linear mixed-effects model predicting spelling of silent final letters

Fixed effects					
Predictor	Estimate [95% CI]	Odds Ratio [95% CI]	SE	Z value	*p*
(Intercept)	0.89 [0.08; 1.69]	2.43 [1.09; 5.43]	0.41	2.17	0.03
Type of silent letter	−1.08 [−2.16; −0.01]	0.34 [0.12; 1.00]	0.55	−1.97	0.049
Group	2.23 [1.68; 2.77]	9.26 [5.37;15.95]	0.28	8.02	< 0.001
Type of silent letter*Group	−0.52 [−1.02; −0.02]	0.60 [1.02; 2.77]	0.26	−2.03	0.043
**Random effec** **ts**					
Random effect		Variance		SD	
Participant (intercept)		0.87		0.95	
Item (intercept)		1.39		1.18	

*CI* Confidence Interval

Significant effects were found for Type of silent letter, Group, and their interaction. In the reference condition (SLFmorpho*control group), spelling accuracy was high, with odds of a correct response exceeding those of an incorrect response (OR = 2.43). Spelling accuracy dropped significantly for SFLcontrol words (OR = 0.34, *p* =.049) and for the dyslexia group (OR = 9.26, *p* <.001). The interaction between Group and Type of silent letter was also significant (OR = 0.60, *p* =.043), indicating that the benefit of morphological predictability (SFLmorpho vs. SFLcontrol) was greater in the dyslexia group than in the control group. In summary, morphologically predictable silent final letters increased spelling accuracy compared to monomorphemic words in both groups. Although the dyslexia group performed overall less well than controls, they benefited more from morphological predictability.

#### Derived words vs. monomorphemic words

In the second set of analyses, the dependent variable was whole-word spelling accuracy across the three conditions (prefixed, suffixed and monomorphemic words). To examine the influence of morphological structure on word spelling, a quadratic contrast (prefixed: 1; suffixed: 1; monomorphemic: −2) was used. Additionally, an orthogonal linear contrast (prefixed: 1; monomorphemic:0, suffixed: −1) was conducted to compare the spelling accuracy of prefixed and suffixed words within each group. Results for the quadratic (i.e., affixed vs. monomorphemic words) and orthogonal (i.e., prefixed vs. suffixed) contrasts are reported in Table 5.

**Table 5:** Generalized linear mixed-effects models predicting spelling of affixed vs monomorphemic words

Model 1. Quadratic contract					
**Fixed effects **					
Predictor	Estimate [95% CI]	Odds ratio [95% CI]	SE	Z value	*p*
(Intercept)	1.13 [0.70; 1.57]	3.11 [2.01; 4.81]	0.22	5.11	<.001
Quadratic contrast	0.40 [0.12; 0.67]	1.49 [1.13; 1.96]	0.14	2.86	.004
Group	−1.68 [−2.00; −1.37]	0.19 [0.14; 0.25]	0.16	−10.49	<.001
Quadratic contrast*Group	0.001 [−0.09; 0.10]	1.00 [0.91; 1.10]	0.05	0.02	.98
**Random effects**					
Random effect		Variance		SD	
Participant (intercept)		0.56		0.75	
Item (intercept)		1.97		1.40	

Model 2. Orthogonal contrast					
Fixed effects					
(Intercept)	1.13 [0.67; 1.59]	3.11 [1.97; 4.91]	0.23	4.87	<.001
Orthogonal contrast	0.28 [−0.22; 0.78]	1.32 [0.80; 2.19]	0.26	1.08	.28
Group	−1.68 [−2.00; −1.37]	0.19 [0.14; 0.26]	0.16	−10.47	<.001
Orthogonal contrast*Group	−0.07 [−0.24; 0.10]	0.93 [0.79; 1.10]	0.09	−0.83	.41
**Random effects**					
Random effect		Variance		SD	
Participant (intercept)		0.56		0.75	
Item (intercept)		2.24		1.50	

*CI* Confidence Interval, Quadratic contrast: monomorphemic words (−2) vs prefixed (+1) and suffixed words (+1) combined; Orthogonal contrast: prefixed words (+1) vs suffixed words (−1), monomorphemic words (0) excluded

The first model included the quadratic contrast and its interaction with Group. Model fit indices are reported for completeness (Log-Likelihood = − 2934.01; AIC = 5880.03; BIC = 5920.05; Deviance = 5357.77). In the reference condition (affixed words*control group), the odds of correctly spelling a word were 3.11 times greater than those of misspelling it, indicating high accuracy in the control group and in the affixed words condition. A significant effect was found for the quadratic contrast, indicating that affixed words (i.e., prefixed and suffixed) were spelled more accurately than monomorphemic words (OR = 1.49, *p* =.004). There was also an effect of Group, with lower accuracy in the dyslexia group compared with controls (OR = 0.19, *p* <.001). Finally, no significant interaction was found between Group and the quadratic contrast (OR = 1.00, *p* =.98), indicating that the effect of morphological construction on spelling accuracy was similar across groups.

The second model included the orthogonal contrast and its interaction with Group. The reference conditions were prefixed words and the control group. Model fit indices are reported for completeness (Log-Likelihood = − 2937.17; AIC = 5886.34; BIC = 5926.36; Deviance = 5357.36). The orthogonal contrast did not reach significance (OR = 1.32, *p* =.28), indicating that spelling accuracy did not differ between prefixed and suffixed words. The effect of Group was significant, showing higher accuracy in the control group than in the dyslexia group (OR = 0.19, *p* <.001). The interaction between Group and the orthogonal contrast was not significant (OR = 0.93, *p* =.41), suggesting that both types of affixation supported spelling equally across groups.

In summary, although spelling accuracy was globally lower in the dyslexia group than in controls, both groups better spelled affixed words—whether prefixed or suffixed— than monomorphemic words. No reliable difference emerged between prefixed and suffixed words, suggesting similar levels of morphological support for both types of affixes. Finally, the absence of interaction effects indicates that adults with dyslexia benefited from morphological structure to the same extent as controls.

### Contribution of morphological awareness to spelling

The second aim of this study was to investigate the contribution of morphological awareness to word spelling in adults with dyslexia. To address this question, hierarchical regression analyses were conducted within the dyslexia group. The aim of these analyses was to examine whether the participants’ level of morphological awareness explained their spelling performance both for affixed words and for a more general measure of spelling (sentence dictation). Phonemic awareness and vocabulary, which are well-established predictors of spelling, were entered at the first step of the regressions, and morphological awareness was added at the second step. As shown in Table 6, morphological awareness made a significant unique contribution to the general spelling score (sentence dictation; *p* =.04, ΔR² = 0.10) and a marginally supported spelling in the SFLmorpho condition (*p* =.08, ΔR² = 0.06). In contrast, morphological awareness did not contribute to the spelling of prefixed or suffixed words (*ps* > 0.10), whereas phonemic awareness significantly predicted performance on prefixed words (*p* =.049, ΔR² = 0.17). None of the other phonemic awareness or vocabulary measures significantly predicted spelling performance.

**Table 6:** Results of hierarchical regression models on the influence of morphological awareness performance on the spelling of morphological information in the dyslexia group

Silent final letter predictable by morphology		β	95% CI		*p*	$\:\varDelta\:$*R*²
			LL UL			
1	Phonemic awareness	0.06	−0.22	0.33	0.68	0.09
	Vocabulary	0.23	−0.04	0.50	0.09	
2	Morphological awareness	0.25	−0.03	0.53	0.08	0.06
Prefixed words						
1	Phonemic awareness	0.27	0.001	0.53	0.049	0.17
	Vocabulary	0.18	−0.08	0.45	0.16	
2	Morphological awareness	0.21	−0.06	0.48	0.13	0.11
Suffixed words						
1	Phonemic awareness	0.08	−0.20	0.36	0.57	0.09
	Vocabulary	0.24	−0.04	0.51	0.09	
2	Morphological awareness	0.16	−0.13	0.44	0.27	0.02
Spelling sentence dictation (numbers of errors)						
1	Phonemic awareness	−0.20	−0.47	0.07	0.14	0.13
	Vocabulary	−0.15	−0.41	0.11	0.27	
2	Morphological awareness	−0.29	−0.56	−0.01	0.04	0.10

*β* standardized beta, *LL* Lower Limit, *UL* Upper Limit, *CI* Confidence Interval

### Compensated profiles with Morpho-phonological dissociation

Finally, exploratory analyses were conducted to examine whether compensated dyslexics among the whole group exhibit different spelling patterns. Participants with dyslexia were divided into two subgroups based on a dissociation index between their phonological and morphological awareness, following the method described by Crawford et al. (2010) and previously used in Cavalli et al. (2017). This case–control dissociation method computes a standardized difference (Z_DCC) between phonological and morphological scores relative to the control group, allowing identification of individuals whose morphological awareness is relatively preserved despite impaired phonological awareness. Individuals with a positive dissociation index (*n* = 10) were classified as “compensated,” while the remaining participants were classified as “non-compensated” (*n* = 43).

Prior to group comparisons, normality of the spelling measures was assessed for each subgroup using Shapiro-Wilk tests. Variables that were normally distributed in both groups were analyzed using independent-samples t-tests, whereas variables violating the normality assumption in at least one group were analyzed using Mann-Whitney tests. Effect sizes were computed using Cohen’s d with Hedges correction for small sample sizes.

Analyses revealed no statistically significant differences between the two groups - compensated individuals (Dys-C) and non-compensated individuals - (Dys-N) across all spelling measures. For general sentence dictation, a Mann-Whitney test showed no difference (*M*Dys-C = 27.90; *M*Dys-NC = 27.98, *W* = 221.50, *p* =.89, *d* = 0.005). Spelling of prefixed words was analyzed using a t-test (*M*Dys-C = 8.50; *M*Dys-NC = 9.41, *t* = 0.72, *p* =.49, *d* = 0.28), and spelling of suffixed words with a Mann-Whitney test (*M*Dys-C = 7.60; *M*Dys-NC = 7.86, *W* = 232.0, *p* =.70, *d* = 0.11). Silent final letters predictable by morphology were analyzed with a Mann-Whitney test (*M*Dys-C = 6.50; *M*Dys-NC = 6.52, *W* = 191.0, *p* =.66, *d* = 0.01).

We then conducted comparisons within each subgroup between the spelling of affixed words (suffixed and prefixed) and monomorphemic words, as well as between spelling of FSLmorpho and FSLcontrol words. Paired t-tests were used for all comparisons, as the differences between paired measures were found to be normally distributed according to Shapiro-Wilk tests. For prefixed versus monomorphemic words, t tests revealed significant differences in both the Dys-C group (*M*prefixed = 8.50, *M*monomorphemic = 5.40), *t* = 3.35, *p* =.008, *d* = 1.06, 95% CI [0.17, 1.95], and the Dys-NC group (*M*prefixed = 9.42, *M*monomorphemic = 4.79), *t* = 11.75, *p* <.001, *d* = 1.79 95% CI [1.30, 2.29]. For suffixed versus monomorphemic words, significant differences were also found in the Dys-C group (*M*suffixed = 7.60, *M*monomorphemic = 5.40), *t* = 2.85, *p* =.02, *d* = 0.90 95% CI [0.05, 1.75], and in the Dys-NC group (Msuffixed = 7.86, *M*monomorphemic = 4.79), *t* = 9.03, *p* <.001, *d* = 1.38, 95% CI [0.95, 1.81]. Finally, spelling accuracy of SFLmorpho words was higher than that of SFLcontrol words in the Dys-C group (*M*SFLmorpho = 6.50, *M*SFLcontrol = 4.50), *t* = 3.16, *p* =.01, *d* = 1, 95% CI [0.12, 1.88], as well and in the Dys-NC group (*M*SFLmorpho = 6.52, *M*SFLcontrol = 4.45), *t* = 5.87, *p* <.001, *d* = 0.91, 95% CI [0.54, 1.27]. The Dys-C group did not appear to rely more on morphological information than the Dys-NC group in spelling words.

## Discussion

Determining whether adults with dyslexia rely on morphological information when spelling is important given their persistent spelling difficulties. This study investigated the role of morphology in spelling among French-speaking adults with dyslexia. We first compared their performance to that of typical readers and then examined the contribution of morphological awareness to their spelling beyond phonological awareness and vocabulary. Analyses were carried out on the whole dyslexia group, with additional exploratory analyses according to their compensation profiles.

Regarding the first research question, adults with dyslexia performed more poorly than controls across all word categories. Nevertheless, both groups spelled words with morphological information more accurately than words without such information. Compared to the control group, the dyslexia group relied more on morphological information when spelling predictable silent final letters, but not when spelling affixed words. Both groups showed better spelling performance for derived words compared to monomorphemic words. While other lexical factors, such as base-word familiarity, morphological family size, or larger orthographic chunks, may also contribute to these effects, the current design does not allow us to disentangle these influences. Within the dyslexic group, exploratory analyses revealed no statistically significant differences were observed between compensated and non-compensated adults on any spelling measure, and both subgroups appeared to benefit similarly from morphological information. Regarding the second research question, morphological awareness significantly predicted general spelling and showed a marginal effect for morphologically predictable silent final letters, but not for prefixed or suffixed words.

### Spelling accuracy of words with morphological information

Our results first confirm that spelling remains a persistent area of vulnerability in university students with dyslexia. Previous studies in Dutch (Callens et al., 2012; Tops et al., 2013, 2014), Spanish (Afonso et al., 2015), and English (Bruck, 1993; Coleman et al., 2009) have already shown that students with dyslexia experience spelling difficulties in addition to reading problems. The present study extends these findings across languages and, for the first time in French, demonstrates that spelling constitutes a major source of difficulty in adults with dyslexia.

Most importantly for our study, our results provide insights into the spelling strategies used by individuals with dyslexia. Since phoneme–grapheme inconsistencies in French can be overcome by relying on morphological information, we examined whether such information could support spelling of individuals with dyslexia. We made this assumption because adults with dyslexia have relatively preserved morphological knowledge (Cavalli et al. 2017; Law et al. 2015; Martin et al. 2014) and make use of morphological information when reading (Cavalli et al. 2017; Lefèvre et al. 2022), thereby supporting the view that the ability to process morphological information emerges, at least in part, independently of phonological abilities.

Our results showed that morphological information helps adult spellers—with dyslexia or not—choose the correct grapheme when phonological cues are insufficient. This confirms findings from studies on skilled adult readers (Heyer, 2021; Treiman et al., 2021) but provides the first such evidence for adults with dyslexia. Although several studies have shown such reliance on morphological information in children with dyslexia (Angelelli et al., 2017; Bourassa et al., 2006; Bourassa & Treiman, 2008; Suárez-Coalla et al., 2017), research on adult spelling remains limited. Yet it is important for advancing our understanding of the mechanisms involved in adult spelling, particularly in individuals with dyslexia. This issue is especially relevant when considering performance on the morphological awareness task, where adults with dyslexia make more errors than controls in deciding whether a word is suffixed. Their orally assessed morphological knowledge is therefore lower than that of controls, suggesting that the strategies they use to select the correct grapheme in spelling do not rely solely on better-developed oral morphological knowledge. Further studies are needed to clarify the mechanisms by which individuals with dyslexia process morphological information during spelling.

A notable finding emerges when comparing adults with dyslexia to controls: the dyslexia group benefited more from morphological information when spelling predictable silent final letters than the control group. This result echoes the study by Quémart and Casalis (2017), which showed that French-speaking adolescents with dyslexia are better able to spell morphologically predictable silent final letters than non-predictable silent letters, in contrast to younger children matched for reading and spelling levels. These findings suggest that this strategy develops relatively early in French-speaking individuals with dyslexia, who must contend with a complex orthographic system that includes a large number of silent final letters. It is, however, possible that this type of strategy is developed through speech-language therapy sessions, which provide methods to circumvent phonological difficulties and support reading and spelling acquisition.

From a theoretical perspective, the observed benefit of morphological predictability in spelling could reflect several cognitive mechanisms. Elbro and Arnbak (1996) proposed that morphological knowledge may help individuals with dyslexia compensate for impaired phonological skills. In this view, when phonological deficits disrupt connections between orthography and phonology, the system may recruit alternative pathways, such as morpho-orthographic mappings - where morphemes are directly linked to their written forms - or semantic routes that support spelling by activating meaning-based information (Harm & Seidenberg, 2004). These strategies could arise from explicit learning or partially automatized processes developed through experience with written language or morphological instruction. Such interpretations are consistent with connectionist models (Harm & Seidenberg, 2004) and the IMP framework (Treiman & Kessler, 2014), which emphasize the interaction between morphology, semantics, and orthography in spelling development.

Finally, the exploratory analyses conducted within the two subgroups of individuals with dyslexia categorized according to their level of compensation, revealed that our sample included only a small number of compensated adults with dyslexia. This may account for the lower performance in morphological awareness compared to the group of typically developing adults, in contrast with the findings reported by Cavalli et al. (2017). Moreover, our exploratory analyses indicate that spelling performance improved in both subgroups in the presence of morphological information. However, this improvement did not differ between compensated and non-compensated individuals, suggesting no additional benefit for the compensated subgroup. Given the small size of the compensated subgroup and the exploratory nature of these analyses, these findings should be interpreted with cautious. More broadly, the well-documented heterogeneity within the dyslexia population makes it difficult to draw firm conclusions about subgroup differences based on limited samples. As such, these results should be considered primarily as hypothesis-generating rather than definitive evidence. This comparison was of interest given that Law et al. (2015) reported higher oral morphological knowledge in compensated dyslexics than in non-compensated dyslexics. However, this additional benefit of compensation does not appear when it comes to utilizing morphological information in spelling. This finding reinforces the idea that spelling performance does not rely solely on oral morphological knowledge, as discussed above. This issue will be further examined in the second part of the Discussion.

### Contribution of morphological awareness on spelling

In the second part of the study, we found that morphological awareness contributed significantly to spelling performance in adults with dyslexia, beyond phonological awareness and vocabulary. However, this contribution was observed only for the overall spelling score (i.e., sentence spelling) and not for the specific scores of silent final letter spelling (despite a trend in this condition), prefixed word spelling, or suffixed word spelling. These findings are consistent with Schiff and Levie (2017), who likewise did not find a significant contribution of morphological awareness to spelling words with varying levels of morphological complexity in Hebrew-speaking adults with dyslexia. The connection between general spelling and morphological awareness might be explained by the fact that an adult with better written language processing skills would be more able to attend to specific features of the language, such as graphemes that don’t follow strict letter-sound correspondences (Sénéchal et al., 2006).

The absence of a significant contribution of morphological awareness to the spelling of morphological information in our study should be interpreted with caution, in light of the particular measure of morphological awareness employed (i.e., suffixation decision task) and potential methodological issues. In the future, multiple measures of morphological awareness should be considered given that it is a multidimensional construct (Apel, 2014; Tighe & Schatschneider, 2015). Indeed, Casalis et al. (2011) reported that the strength of correlations between morphological awareness and spelling varied depending on the specific task used (analogy task with a stable relationship between base and derived vs. unstable relationship). Statistical constraints may also account for our results. In the control group, some spelling measures, particularly SFLmorpho and affixed word conditions, were near ceiling. This restricted variance may have contributed to the absence of significant correlations between morphological or phonological awareness and spelling performance. However, even for spelling measures without ceiling effects, correlations with morphological and phonological awareness remained weak, suggesting that limited variance alone does not fully account for these null associations. Overall, the general spelling score showed greater variability than isolated word spelling scores, leaving more room for significant correlations to emerge.

### A perspective on morphological knowledge and written language processing in dyslexia

From a theoretical perspective, the relationship between morphological knowledge and written language processing remains a matter of debate, as the mechanisms linking oral and written language are not always clearly understood. Although a specific link has been reported between morphological knowledge and reading (e.g., Levesque et al., 2021), other studies have failed to demonstrate a specific contribution of morphological knowledge to written language, instead pointing to a more general relationship between oral and spelling (e.g., Casalis et al., 2011).

In our study, morphological knowledge assessed through a morphological awareness task accounted for certain aspects of spelling performance in adults with dyslexia, but not all. Recent theories of spelling acquisition suggest that the ability to process morphological information during spelling relies both on strategies grounded in oral morphological knowledge and on statistical learning derived from exposure to print (IMP framework, Treiman & Kessler, 2014). Thus, improvements in spelling performance require both an awareness of the morphological structure of words in oral language and extensive exposure to written language. In line with this, connectionist models of reading and spelling also propose that spellers gradually develop knowledge of the morphological and orthographic patterns of their language as they encounter orthographic inconsistencies (e.g., Harm & Seidenberg, 2004; Plaut et al., 1996). Although morphological knowledge is less developed in individuals with dyslexia compared to age-matched peers, they nonetheless appear to rely more heavily on morphological regularities than their peers when reading (Cavalli et al. 2017; Georgiou et al. 2023; Law et al. 2018; Lefèvre et al. 2022) but also when spelling, as evidenced here. As a consequence, the difficulty adults with dyslexia experience in developing and linking their oral morphological knowledge to spelling confirms that morphological awareness training is a promising target for intervention (see Ardanouy & Quémart, 2025 for a similar proposition).

Interestingly, phonological awareness showed only a limited effect—restricted to prefixed word spelling—consistent with findings that its role diminishes in adulthood (Roman et al., 2009), while morphological awareness becomes a more reliable predictor in poor-reading adults (Cheema et al., 2023) or adults with dyslexia (Law et al., 2015). Overall, morphological strategies may provide more stable support for orthographic accuracy in adults with dyslexia, particularly when dealing with irregularities, whereas phonological skills appear more task-dependent (Law et al., 2015).

### Educational implications

From a developmental perspective, morphological knowledge has been shown to support spelling from an early age. However, their interest may increase over time, as learners gain more exposure to written language and as phoneme-grapheme correspondences become less effective, particularly in languages with orthographic inconsistencies. In adolescence and adulthood, individuals are also more cognitively equipped to manipulate abstract linguistic structures, making explicit instruction in derivational morphology especially valuable at these stages. Our findings argue for the integration of morphological instruction into spelling interventions targeting older adolescents and adults with dyslexia. In our sample, adults with dyslexia demonstrated persistent difficulties in both morphological awareness and the spelling of morphologically complex words, including prefixed and suffixed forms as well as words with silent final letters predictable by morphology. Based on these findings, we recommend prioritizing the following instructional components: (1) activities to strengthen morphological awareness; (2) training in the spelling and the meaning of suffixes and prefixes within different words, with greater emphasis on suffixes, which are more difficult to master, probably because of their lack of connection with semantics; (3) training on the morphological predictability of silent final letters particularly the use of morphologically related words (such as the feminine form of a word or words from the same morphological family), using a variety of final letters, not just ‘t’, which is one of the most frequent final letter.

### Limitations and perspectives

One limitation is that this research focused on university adults with dyslexia, but it is known that only a small number of people with dyslexia attend university (only 2% attend university while the prevalence in the general population is around 5 to 10%; Horn & Berktold, 1999). Therefore, it would be important to explore spelling among non-university adults to see if our results are similar. It could be the case that the morphological awareness of non-university adults with dyslexia may be less well-developed (due to a less developed vocabulary from reduced exposure to written language), resulting in lower benefit from the use of morphological strategies. Another important limitation is that we used only a single suffix detection task to assess morphological awareness, because this task already existed and had previously been used with an adult population with dyslexia (Cavalli et al. 2017). However, morphological awareness is a multidimensional skill that would ideally be assessed using multiple tasks (e.g., prefixes vs. suffixes, explicit vs. implicit tasks, inflection vs. derivation). Therefore, the present findings should be replicated using a broader set of tasks to more comprehensively assess morphological awareness.

In terms of future perspectives, given the persistent spelling difficulties of adults with dyslexia and their motivation to improve their spelling, as was evident in the participants in this study, it seems opportune to propose interventions focused on derivational morphology in adults with dyslexia. The adult population with dyslexia tends to be understudied, despite the promising results of interventions that have been conducted with this population (Bar-Kochva et al., 2020; Gray et al., 2018). In addition, these findings may also be relevant for other languages, particularly those with morphophonemic orthographies (e.g., English, Dutch), where morphological information plays a key role in resolving spelling ambiguities.

In conclusion, our results underscore that morphological information can provide a valuable source of support for spelling in adults with dyslexia. Despite reduced morphological awareness, they are able to draw on morphological information to improve spelling accuracy, although the extent of this benefit varies across word types. Taken together, these results advance our understanding of the spelling profile of adults with dyslexia.

## Supplementary Information

Below is the link to the electronic supplementary material.

Supplementary Material 1

Supplementary Material 2

## Data Availability

The data are available from the authors upon request.

## Footnotes

1 Graphotactic patterns refer to language-specific regularities and constraints that govern which letter combinations are legal or typical in a writing system.

2 The term “inconsistent monomorphemic word” can be defined as a word that contains only one morpheme—i.e., it is not derived—but includes at least one phoneme-to-grapheme correspondence that is multiple or infrequent, which can lead to spelling errors if only a phonological strategy is used.
