# Supplementary material for: Morphological knowledge and spelling in French-speaking adults with developmental dyslexia
Source: Ann Dyslexia. 2026 May 19;76(3):511–37. doi: 10.1007/s11881-026-00373-2 (PMC13759549; doi:10.1007/s11881-026-00373-2)
Supplement: Supplementary file 1 [file 11881_2026_373_MOESM1_ESM.docx]

**Appendix A**

*List of stimuli for words with final silent letters*

|  | Silent letter predictable by morphology | Non-predictable silent letter |
| --- | --- | --- |
| 1 | Rabai**s** (discount) | Anet**h** (dill) |
| 2 | Nauséabon**d** (putrid) | Pistole**t** (pistol) |
| 3 | Céliba**t** (single status) | Berlingo**t** (hard candy) |
| 4 | Astico**t** (maggot) | Perdri**x** (partridge) |
| 5 | Matela**s** (mattress) | Velour**s** (velvet) |
| 6 | Rebon**d** (rebound) | Abbay**e** (abbey) |
| 7 | Confi**t** (candied) | Artichau**t** (artichoke) |
| 8 | Fayo**t** (crawler) | Caddi**e** (cart) |
| 9 | Accro**c** (snag) | Courrou**x** (anger) |
| 10 | Confu**s** (embarrassed) | Camember**t** (camembert) |
