# Supplementary material for: Morphological knowledge and spelling in French-speaking adults with developmental dyslexia
Source: Ann Dyslexia. 2026 May 19;76(3):511–37. doi: 10.1007/s11881-026-00373-2 (PMC13759549; doi:10.1007/s11881-026-00373-2)
Supplement: Supplementary file 2 [file 11881_2026_373_MOESM2_ESM.docx]

**Appendix B**

*List of stimuli for prefixed words, suffixed words and monomorphemic words*

|  | Prefixed words | Suffixed words | Monomorphemic words |
| --- | --- | --- | --- |
| 1 | **Mé**disant (slanderous) | Absten**tion** (abstention) | Grommeler (to mumble) |
| 2 | **In**nombrable (countless) | Immond**ice** (filth) | Harassé (exhausted) |
| 3 | **Al**léger (to lighten) | Marronn**ier** (horse chestnut tree) | Serpillière (mop) |
| 4 | **Ir**rationnel (irrational) | Authentic**ité** (authenticity) | Labyrinthe (labyrinth) |
| 5 | **Im**meuble (building) | Echafaud**age** (scaffolding) | Friction (friction) |
| 6 | **Im**modéré (immoderate) | Sauter**elle** (grasshopper) | Chorale (choir) |
| 7 | **Dé**dommager (compensate) | Chaot**ique** (chaotic) | Chrétien (Christian) |
| 8 | **Sur**réaliste (surrealist) | Exig**ence** (demand) | Professeur (professor) |
| 9 | **Em**boîter (to slot) | Insoupçonn**able** (above suspicion) | Blasphémer (to blaspheme) |
| 10 | **A**baisser (to lower) | Combust**ible** (combustible) | Suspense (suspense) |
| 11 | **Em**barcation (craft) | Coïncid**ence** (coincidence) | Auréole (aureole) |
| 12 | **Més**entente (disagreement) | Coll**ier** (necklace) | Acolyte (acolyte) |
| 13 | **Sur**enchère (higher bid) | Tyrann**ique** (tyrannical) | Référendum (referendum) |
| 14 | **Dés**œuvré (idle) | Substanti**el** (substantial) | Balbutier (to stammer) |
| 15 | **Bi**coque (catamaran) | Gaie**ment** (happily) | Couffin (carrycot) |
| 16 | **In**descriptible (indescribable) | Narratr**ice** (narrator) | Alligator (alligator) |
| 17 | **Im**migré (migrant) | Pérenn**ité** (durability) | Faïence (earthenware) |
| 18 | **Im**mobilisé (immobilized) | Pays**age** (landscape) | Cacahuète (peanut) |
